# A Financing Model to Solve Financial Barriers for Implementing Green Building Projects

**DOI:** 10.1155/2013/240394

**Published:** 2013-11-24

**Authors:** Sanghyo Lee, Baekrae Lee, Juhyung Kim, Jaejun Kim

**Affiliations:** ^1^Sustainable Building Research Center, Hanyang University, 1271 Sa3-dong, Sangnok-gu, Ansan-si, Kyunggi-do 426-791, Republic of Korea; ^2^Department of Architectural Engineering, Hanyang University, 17 Haengdang-dong, Seongdong-gu, Seoul 426-791, Republic of Korea; ^3^Department of Sustainable Architectural Engineering, Hanyang University, 17 Haengdang-dong, Seongdong-gu, Seoul 426-791, Republic of Korea

## Abstract

Along with the growing interest in greenhouse gas reduction, the effect of greenhouse gas energy reduction from implementing green buildings is gaining attention. The government of the Republic of Korea has set green growth as its paradigm for national development, and there is a growing interest in energy saving for green buildings. However, green buildings may have financial barriers that have high initial construction costs and uncertainties about future project value. Under the circumstances, governmental support to attract private funding is necessary to implement green building projects. The objective of this study is to suggest a financing model for facilitating green building projects with a governmental guarantee based on Certified Emission Reduction (CER). In this model, the government provides a guarantee for the increased costs of a green building project in return for CER. And this study presents the validation of the model as well as feasibility for implementing green building project. In addition, the suggested model assumed governmental guarantees for the increased cost, but private guarantees seem to be feasible as well because of the promising value of the guarantee from CER. To do this, certification of Clean Development Mechanisms (CDMs) for green buildings must be obtained.

## 1. Introduction

An overwhelming body of scientific evidence now clearly indicates that climate change is a serious and urgent issue. The Earth's climate is rapidly changing, mainly as a result of increases in greenhouse gases (GHGs) caused by human activities [1]. Among the GHGs, excessive emissions of carbon dioxide from the use of fossil fuel for energy generation have created unprecedented environmental pollution and health risks [2]. Sustainable and renewable energy technologies are solutions for reducing the use of fossil fuel and for meeting energy demands [3]. In particular, construction products constitute a major problem to solve in terms of their large amount of energy consumption, and thus minimizing their negative impacts on the environment is an important issue [4]. From this perspective, green buildings, to which sustainable and renewable energy technologies are applied, minimize the impacts of buildings on the environment [5].

In tune with the global trend and a growing interest in green buildings for greenhouse gas energy reduction, the government of The Republic of Korea began to emphasize green growth as its national growth paradigm. However, a green building generally has greater initial construction costs than other buildings [6]. With uncertainties in the future value of a project, increased costs may have negative effects on financing. Therefore, the government needs to take the necessary measures for smooth financing to vitalize green building projects. In other words, the government needs to share the risks with the private sector or to provide means to hedge the risks. For example, Public-Private Partnership (PPP) is a way to share risks through governmental guarantees [7]. In this way, using governmental guarantees can support private financing for green building projects. 

However, when the government participates in green building projects as a party to risk sharing, returns on risks are necessary. This is because the government is so sensitive to the risk of project failure that it can be passive in providing guarantees.

This study suggests a financing model for green building projects with governmental guarantees based on Certified Emission Reduction (CER), which is an emerging trend in environment finance. 

For analysis, this study utilized energy savings (%) and increased costs (US$/3.3 m^2^) data of three residential building cases having different equipment for energy saving. Thus, this study assumed three cases of the residential building project having various equipment. Cash flow was estimated with the assumption that variables, except construction costs and financing costs resulting from increased construction cost for the residential building project, are the same.

The financing model suggested in this study can be tested by estimating the time to retrieve the value of governmental guarantee through the CER transaction. Thus, the values of governmental guarantee and CER need to be estimated. First, the value of governmental guarantee was estimated using the concept of a put option. To estimate the value of CER, the carbon emissions of the residential building project were estimated using the equation suggested by the IPCC. The estimated carbon emissions of the residential building project were applied to the three cases of energy savings (%) to obtain the CO_2_ emission reduction of each case. For the price of CER, the European Climate Exchange (ECX) data from January 9, 2006, to November 19, 2010, were used. This study set the greatest price of unit CER as the best scenario, the smallest price as the worst, and the average price as a moderate scenario. Thus, by combining the CO_2_ emission reduction and CER of each scenario, the value of CER for each scenario was obtained. Using the value of CER estimated for each scenario and the governmental guarantee, the payback period was obtained and the feasibility of the financing model was tested. 

## 2. Background 

### 2.1. Literature Review

There is a growing interest in green buildings as the great effect of building activities on the environment is gaining attention. Thus, the review of the literature on green building revealed that there have been a number of studies on various subjects such as assessment tools [8], occupant satisfaction [9], energy ratings [10], design [11], and barriers to implementing green building [12, 13]. 

Green building is a new business model resulting from global awareness of the environment, and various ways forward for success need to be reviewed. From this perspective, barriers to implementing green building need to be considered not only from the technical point of view but also from the business point of view. 

Zhang et al. [12] investigated the effects of increased costs and various barriers when green elements were applied to a construction project. They demonstrated that construction cost increases for all cases in which green elements were applied. Furthermore, ten barriers were selected and a survey was conducted on them, resulting in the identification of construction costs as the greatest barrier. Intrachooto and Horayangkura [13] suggested that the application of a new technique to a construction project incurs increased costs and results in increased risk and uncertainty from the perspective of business feasibility. This problem constitutes a financial barrier to implementing green building projects. Accordingly, the increased costs can be a barrier to financing. Thus, this study suggested a financial model to overcome the financial barriers resulting from the additional costs. 

### 2.2. Financial Barriers for Implementing Green Building Projects

Investors' primary goal is to make benefits or to get a return on investment. This rate of return can be determined by three different indicators: payback time, the return on investment, and the internal rate of return [6]. These indicators are also considered three downsides of a green building project. Concretely, investment in a green building project can be returned in about 7-8 years because the benefit of energy saving occurs in the operation stage [14]. As investors prefer a short-term payback time [15], they are relatively passive in investing in green building projects with their long-term payback time. Moreover, long-term payback time tends to be accompanied by uncertainties regarding project success, exposing the projects to greater risks. On that account, if the interest rates increased, damage from the rising project costs cannot be avoided in spite of well-disposed investors [6]. In the end, a green building project has barriers in private financing due to the long-term payback time, relatively high interest rates, and increased initial costs. Therefore, to launch green building projects, governmental supports are necessary to remove such financial barriers.

### 2.3. Green Building in the Republic of Korea

The Republic of Korea was ranked the 10th country in carbon emissions in 2008 according to the announcement by the International Energy Agency (IEA) [16]. After the Kyoto Protocol mandated reduction of greenhouse gas (GHG) emissions, a green growth policy was set by the government of The Republic of Korea in order to meet the requirement. 

To reduce CO_2_ emissions, building energy consumption needs to be reduced. According to the IEA, buildings account for 40% of the world's final energy consumption and 24% of CO_2_ emissions. The IEA referred to commercially available, renewable technologies as a means to improve the efficiency of buildings' energy systems and to reduce a large portion of the energy consumption [6]. Further, as reported by statistics from Korea Energy Economics Institute (KEEI), buildings accounted for about 22% of the total energy consumption and 25% of the total CO_2_ emissions as of 2007 in The Republic of Korea [17]. From this perspective, it is expected to the substantial effect on decrease in CO_2_ emission, aggressively utilizing green building projects is an urgent matter in the Republic of Korea. 

### 2.4. Environment Finance

Environment finance encompasses all market-based instruments designed to deliver environmental quality and to transfer environmental risks [18]. In this respect, the financing structure model of this study would fall under environment finance. 

Due to the recent growing global interest in the environment, various environmental issues are changing and shaping investment markets and capital flows [19]. Thus, researches on environment finance have been performed actively. Peszko and Zylicz [20] stated that the demand for environment finance is influenced by environmental policy or a company's financing capability, and thus governmental support needs to be thoroughly reviewed. They specifically referred to measures that effectively attract private investment in environment finance [20]. Actually, Branker and Pearce [21] mentioned the important role of government support in large-scale, thin film solar photovoltaic manufacturing and the feasible financial return on it. Green building projects may have difficulties in obtaining private funding because they have large initial investments to improve energy efficiency, compared to other building projects. Therefore, governmental guarantees, to share the risk of project default with the private sector, can be effective for the efficient funding of green building projects [21]. 

In addition to the government role in environment financing, carbon has attracted attention as an asset in this field. Chaurey and Kandpal [22] measured the carbon mitigation potential through Solar Home Systems (SHS) and, based on this, studied the effect of carbon finance. In other words, they regarded carbon, a representative GHG, as a kind of asset and, using this, tested the effectiveness of their finance model [22]. Lewis [23] analyzed how carbon finance currently plays a role and how it can be reformed afterward in the developing world [23]. An emerging trend in environment financing is a trading market for CER given that carbon, generated from the use of fossil fuel, has a great effect on the natural environment. 

The financing model of this study would facilitate the sharing of risks by using governmental guarantees. The government could continue to provide guarantees whether there were returns on the risks. Namely, CER is an asset from the perspective of environment finance because of the existence of a trading market. Consequently, the government could see CER, predicted carbon emission reduction through the green building project, as a return for the guarantee.

### 2.5. Real Options Theory

This study suggests a financing structure model for green building projects having a governmental guarantee agreement based on carbon emissions reduction by diminishing energy consumption. The government may face financial difficulties if it shares risk for green building projects with no strings attached. Governmental guarantees and expected profits from CER need to be compared to test the feasibility of the financing structure model. Thus, this study evaluated the value of governmental guarantees using real options, which can consider uncertainties involved in the success of the project.

An option is a security given the right to buy or sell an asset, subject to certain conditions, within a specified period of time [24]. In financial market, the most common types of options are a call option and a put option. A call option gives the owner the right to buy a stock at a predetermined exercise price on a specified maturity date. Conversely, a put option gives its owner the right to sell the stock at a fixed exercise price [25]. The option pricing theory has been applied in the evaluation of nonfinancial assets or real investments; researchers also called it real options. This dynamic pricing process overcomes difficulties in the discounting approach, such as the Net Present Value (NPV) method, and computes the value of a strategic investment more realistically [26]. 

**Figure 1:**
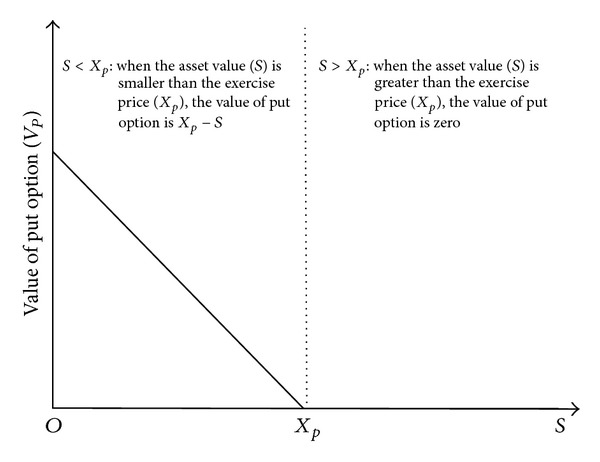
The value of put option depending on the change of the asset value.

To measure the value of governmental guarantee, the value of a put option used is described as in Figure 1. In other words, when the asset value is greater than the exercise price (*S* > *X*_*p*_), a put option does not have any value because selling the asset for its value is better. However, when the asset value is smaller than the exercise price (*S* < *X*_*p*_), selling the asset for the exercise price is much better. Here, the value of a put option is *X* − *S*. 

Previous studies on real options show that real options are used not only for assessing the value of various tangible assets, such as a technology investment [27], infrastructure investment [28], and mine production [29], but also for assessing the value of contracts to parties such as material procurement contracts [30] and guaranteed contracts [31].

This study suggests a financing structure model for green building projects having a governmental guarantee agreement based on carbon emissions reduction by diminishing energy consumption. The government may face financial difficulties if it shares risk for green building projects with no strings attached. Governmental guarantees and expected profits from CER need to be compared to test the feasibility of the financing structure model. Thus, this study evaluated the value of governmental guarantees using real options, which can consider uncertainties involved in the success of the project.

## 3. Suggestion about Financing Model for Green Building Projects

### 3.1. Proposed Framework

**Figure 2:**
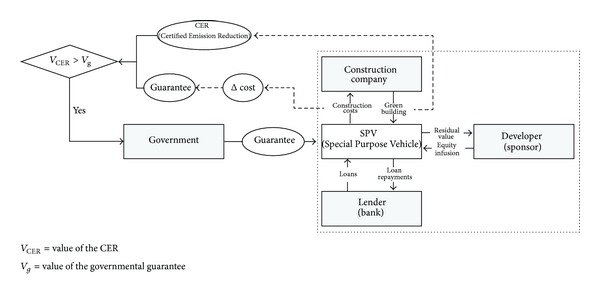
Proposed financing model for facilitating Green Building.

As mentioned above, a green building project has increased initial construction costs. In the end, it negatively works as a barrier to attracting private funding due to uncertainties about future project value. Thus, governmental support for gathering private funding for green building projects is necessary and this study suggests a financing model with governmental guarantees for the increased initial construction costs.  Figure 2 shows the conceptual diagram for the financing model in this study.

Existing building projects are funded by project financing. Special Purpose Vehicle (SPC), invested by a developer (sponsor), raises funds from lenders using future cash flow of the project and makes construction contracts with a construction company. A green building project generally has greater construction costs than other building projects. If the government provided guarantees for the increased construction costs, lenders could remove additional uncertainties caused by them. However, the governmental guarantees entail governmental participation in the project, and thus, the government is also directly affected by the risk of project failure. Further, if the government has to provide guarantees only because a project is a green building project, the government may have to bear a considerable burden. As a result, the government needs to secure return for its guarantee. This return must be defined in terms of assets for which a trading market exists. From this perspective, this study defined CER, benefits from energy saving in the operational stage, as a return on a green building project. Actually, CER is being traded as an asset through the Clean Development Mechanism (CDM). As a result, after assessing the value of governmental guarantees for green building projects and comparing it to the value of CER, governments could determine whether to provide guarantees.

### 3.2. Concept of Valuing Governmental Guarantee Using Real Options

Guarantee contract is one of the safety provision to retrieve the investment. Accordingly, the value of guarantee increases when the project fails. This is very similar to the concept of a put option. There are a number of studies, which have applied the concept of put options to the evaluation of various payment guarantees such as valuation of deposit insurance by Federal Deposit Insurance Corporation (FDIC) [32] or Federal Loan Guarantees to Corporations [33]. The process of this study, to value the governmental guarantee using the option theory, is as follows: total costs (*C*_*t*_) for a green building project can be defined as the sum of costs (*C*_*s*_) for a building of the same size and additional costs(Δ*C*_*a*_) for the green building. Therefore, one has

(1) $$\begin{array}{c} C_{t}=C_{s}+\Delta C_{a}. \end{array}$$

Here, liabilities (*L*) can be defined as total costs (*C*_*t*_) less equity (*E*) invested by a developer (sponsor) as follows:

(2) $$\begin{array}{c} L=C_{t}-E. \end{array}$$

For project financing in The Republic of Korea, equity (*E*) is generally used to purchase land for the project and liabilities are generally used for actual construction. In other words, additional costs (Δ*C*_*a*_), for the green building project, are lowly relevant to equity (*E*). Thus, (2) can be simplified as follows:

(3) $$\begin{array}{c} L=\left(C_{s}-E\right)+\Delta C_{a}. \end{array}$$

**Figure 3:**
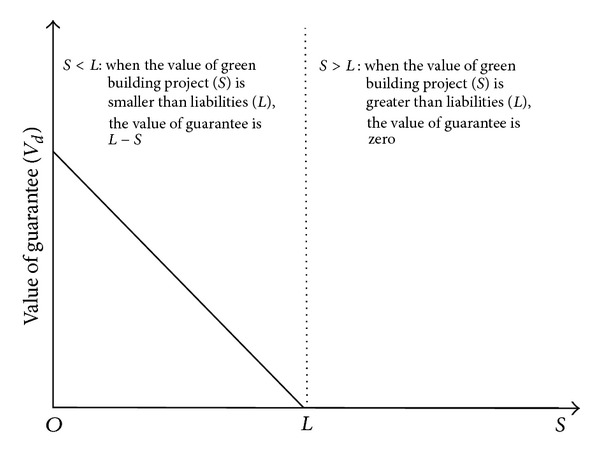
The value of guarantee depending on the change of a project value.

Let us assume that the value of the green building project is *S* and the guarantee for liabilities (*L*) was provided. This means even if *S* is less than *L*, lenders can get *L* through the guarantee. In other words, the value of the guarantee changes, like the value of the put option mentioned above.  Figure 3 shows that when *S* is greater than *L* (*S* > *L*), *L* can be retrieved by *S* and the value of the guarantee is zero. However, when *S* is smaller than *L* (*S* < *L*), *L* cannot be retrieved. However, through the guarantee, even if *S* is smaller than *L*, *L* can be retrieved, and thus the value of the guarantee increases as *S* decreases, being *L* − *S*. Thus, *L* is the same as the striking price of the put option (*X*_*p*_). The value of the put option (*V*_*p*_) is the value of the guarantee (*V*_*d*_).

Accordingly, the value of the governmental guarantee (*V*_*g*_) for the additional costs (Δ*C*_*a*_) is as follows:

(4) $$\begin{array}{c} V_{g}=V_{d}\times \left(\frac{\Delta C_{a}}{L}\right). \end{array}$$

## 4. Applications

### 4.1. Data Set

The aim of this section is to test the financing model for a green building project having a governmental guarantee agreement based on CER, which is obtainable from energy saving. Three cases for a residential building project having different equipment for energy saving were analyzed.  Table 1 shows each case's equipment for energy saving, energy savings (%), and increased costs (US$/3.3 m^2^). The residential building project of this study was assumed to have three kinds of equipment. 

Variables, except construction costs and financial costs increasing construction costs for the residential building project, were assumed to be the same for cash flow as presented in Table 2. Using the cash flow, the value of the governmental guarantee was assessed. 

Cash flow for each case and the details for variables estimating the value of governmental guarantee are as follows.

In Table 3, the underlying asset value (*S*_0_) is the present value of income and, in this study, was estimated as US$221.76 million. As described above, the striking price (*X*_*p*_) is the same as the loan, and thus, for each case, it was estimated at US$206.98 million, US$207.49 million, or US$207.90 million. Sales income was determined by sales price and sales rate. In this study, house price index data from March 2004 to October 2010 and sales rates of 50%~100% were used and combined to measure volatility (*σ*). Volatility (*σ*) was about 25.6% interest for a three-year maturity government loan and public bonds were used as a risk-free rate (rf), which was 5.36%. The time step of a half-year unit was used. 

Thus, up-step size (*u*) of 1.198, down-step size (*d*) of 0.834, and risk-neutral probability (*p*) of 0.529 were obtained. Using these, the put option value for liabilities was estimated and using (4) the value of the governmental guarantee for the increased construction costs for the green building project was obtained. 

CER is currently traded on the EU Emission Trading Scheme (EU-ETS) in the EU and on Chicago Climate Exchange (CCX) in the USA. CER prices from January 9, 2006, to November 19, 2010, at the European Climate Exchange (ECX) were used in this study.

### 4.2. Valuing the Governmental Guarantee

To test the financing model for a green building project supported by a governmental guarantee agreement based on CER, the value of the governmental guarantee was obtained using the option theory. As described above, the put option value was obtained to calculate the guarantee value. Building project data are discrete as monthly data, unlike continuous data for other financial assets. Thus, a binomial lattice model was used to estimate the option value, and not the Black-Scholes model, assuming continuous time flow.  Table 4 shows the values of the governmental guarantee for each case, obtained using (4).

As Table 4 shows, additional equipment for energy saving leads to increased construction cost and increased value of the governmental guarantee. By comparing the obtained value of the governmental guarantee to the value of CER, the feasibility of the financing model for a green building project was tested.

### 4.3. Comparison of the Values of the Governmental Guarantee and CER

First, CO_2_ emissions were estimated for the project to compare the values of the governmental guarantee and the CER. The CO_2_ emissions were estimated using (5) suggested by the IPCC, and Table 5 shows the results. One has

(5) CO2  emission (ton  CO2) =Energy  consumption (TOE)  ×Carbon  emission  factor  (ton C/TOE)  ×Burning  rate (%)×(4412).

The result of energy saving (%) of each case applying into the residential building project and value of governmental guarantee in Table 4 is following Table 6.

CER prices at European Climate Exchange (ECX) from January 9, 2006, to November 19, 2010, were used in this study. This study used the highest price of unit CER as the best scenario, the lowest price of unit CER as the worst scenario, and the average of both as the moderate scenario. As a result, Table 7 shows payback periods for the value of governmental guarantee using CO_2_ emission reduction, guarantee value, and CER price for each case and each scenario. 

As in Table 7, as the CER unit price goes down, the total CER goes down and the payback period becomes longer. Further, as energy saving (%) increases, total CER increases as well. However, as construction costs increase, the value of the governmental guarantee increases as well. However, the increase in the value of the governmental guarantee is greater than that in the total CER by increased energy savings (%), and thus, as energy savings (%) increase, the payback period becomes longer. If the efficiency in energy saving for equipment is improved, the payback period would become shortened. Given the forty-year limitation for legal reconstruction in The Republic of Korea, the longest payback period of 7.55 years for the worst scenario shows that the financing model of this study is feasible for implementing actual green building projects. 

### 4.4. Discussion

The financing model of this study assumed governmental guarantees for the increased cost, but private guarantees seem to be feasible as well because, in return for the guarantee, the value of the guarantee can be obtained through CER. To vitalize this financing model, private investments have to become active in the market system. Nevertheless, this study assumed the governmental participation to test the feasibility of the financing model. Showing the market the feasibility of the financing model, involving governmental participation in a green building projects, may induce private investments. In addition, given the national priority to meet CO_2_ emission reduction targets imposed by the climate accord, the government needs to take the initiative and to play an important role in implementing the financing model. To vitalize the financing model in the market, CDM certificates need to become vitalized as well. Current CDM certified projects are mostly plant projects. However, given that buildings account for 40% of the total final energy consumption and 24% of CO_2_ emissions in the world according to IEA, active implementation of green building projects will be very effective in CO_2_ emission reduction. From this perspective, the CDM certificate system for green building projects needs to become active. If it would be active, various financing methods can be developed based on the financing model of this study and CER.

## 5. Conclusions

The Kyoto Protocol, which went into effect in February 2005, has led to global efforts to reduce CO_2_ emissions. Especially in The Republic of Korea, the government has set green growth as its paradigm for national development and there is a growing interest in greenhouse gas energy reduction for green buildings. However, green buildings have increased initial construction costs and there may be difficulties in financing for green building projects. To deal with this problem, this study suggests a financing model for a green building project having a governmental guarantee based on CER obtainable from energy saving. In other words, by providing a governmental guarantee for green building projects, the government can be directly affected by the risk of project failure. If the government provides a number of guarantees for green building projects, the government's financial status could be affected. Accordingly, this study used CER in actual trading markets as a return for the guarantee. 

By testing the suggested financing model using the combination of degree of energy saving and CER price scenarios, the payback period for the worst scenario was about 7.55 years. Comparing this to forty years of remodeling limitation, the financing model of this study turned out to be feasible for actual green building projects. 

The financing model of this study used the governmental guarantee for the increased cost. However, there is a return for the guarantee through CER, and thus, private guarantees are feasible as well. Therefore, the financing model of this study can be used in the private sector as well. 

However, for the application of the financing model suggested in this study, the CDM certificate system needs to be implemented first. To trade CER, the corresponding project must be CDM certified. Actually, most CDM certified projects are plant projects. However, given that buildings account for 40% of the final energy consumption and for 24% of CO_2_ emissions, the CDM certificate system needs to be applied to buildings as well.

Most financing models have a risk as a result of uncertainties in future asset values. The financing model of this study also has such risk even if the value of the guarantee was estimated in consideration of such uncertainties using the real options theory. If a project fails, additional costs greater than the estimated value of the guarantee need to be paid, and, in this case, the payback time can be greatly lengthened. From this perspective, even if the value of the guarantee can be retrieved through the CER transaction, ways for reducing the risk need to be prepared.

## Figures and Tables

**Table 1:** Overview of three residential building projects applying different energy reduction items.

Category	Items		Equipment			Energy saving (%)			Additional costs (US$/3.3 m^2^)		
			Case 1	Case 2	Case 3	Case 1	Case 2	Case 3	Case 1	Case 2	Case 3
		Change of the insulating materials	Neopor thermal insulation (0.032 W/m^2^K)				4.00			1.45	
		Change of the insulation thickness of the bedroom and balcony	0.156 W/m^2^K/170 mm				7.80			5.70	
	Application of the insulating materials	Change of the insulation thickness of the balcony	0.432 W/m^2^K/55 mm						
		Change of the insulation thickness of the ground and top floor	—	0.195 W/m^2^K/0.175 W/m^2^K 150 mm/150 mm (65 + 85)		—	1.50		—	0.32	
		Reinforcement of the pipe insulation	—	Elastomeric flexible cellular insulation		—	0.50		—	2.12	
IR		Change of windows of the bedroom	0.97 W/m^2^K/22 mm + 16 mm (double glazing) (PVC290)				3.40			2.62	
		Change of windows of the balcony	Dry: PVC170 (L/S), wet: PVC130 (S/D)				2.20			1.52	
		Change of windows of the living room	1.51 W/m^2^K/52 mm (triple glass (R183))	1.18 W/m^2^K/52 mm (triple glass (PVC220))		4.30	8.30		2.66	4.02	
	Application of hi-per windows	Reinforcement of thermal performance of the windows	—		1.51 W/m^2^K (double window) (PVC16 mm + PVC16 mm)	—		1.40	—		4.23
		Reinforcement of thermal performance of the front door	—		0.99 W/m^2^K (insulation door)	—		0.50	—		1.09
		Reinforcement of thermal performance of the door to balcony	—		2.16 W/m^2^K (wood door)	—		1.30	—		1.36

	Boiler	Boiler	Condensing boiler				8.94			0.36	
	Application of the private high efficiency equipment	High efficiency lamps (bedroom)	FPL32W				1.05			0.03	
		High efficiency lamps (bathroom)	FL28W				0.14			0.03	
HE		High efficiency lamps (front door, balcony)	LED21W/EL15W				1.04			0.58	
		Ventilation system	—	Heat recovery ventilator + heat exchanger		—	7.00		—	5.30	
	Application of the public high efficiency equipment	High efficiency lamps (basement garage)	FL29W				1.23			0.03	
		Electric transformer	High efficiency electric transformer				1.53			0.84	

RE	Photovoltaic system		0.06 kw/housing unit				3.2			2.78	

O	Water treatment system		Water transportation pump				0.12			0.02	
	LED (light-emitting diode) signs		LED				0.46			0.11	

Total						33.96	46.96	50.16	18.71	27.53	34.50

IR: insulation reinforcement.

HE: high efficiency equipment.

RE: renewable energy.

O: other.

**Table 2:** Cash flow for each case.

Project	NPV	Costs				Income
		Loans	Direct costs	Financial costs	Sum of other costs	
Base project	6.76	205.89	55.70	24.32	145.03	249.59
Project applied case 1	5.48	206.98	57.03	24.42		
Project applied case 2	4.87	207.49	57.65	24.47		
Project applied case 3	4.39	207.90	58.14	25.51		

Unit: million US$.

**Table 3:** Parameters to estimate the value of governmental guarantee.

Parameters	Value
Underlying asset value (*S*_0_, million US$)	US$221.76
Time step (dt)	1/2 year
Volatility (σ)	25.6%
Risk-free rate (rf)	5.36%
Up-step size (*u*)	1.198
Down-step size (*d*)	0.834
Risk-neutral probability (*P*)	0.529
Striking price (*X*_*c*_, million US$)	
Application of case 1	US$206.98
Application of case 2	US$207.49
Application of case 3	US$207.90

**Table 4:** Value of guarantee for each case.

Classification	Option value (*V*_*d*_)	Loan (base)	Loan (*L*)	Additional costs (Δ*C*_*a*_)	Guarantee value (*V*_*g*_)
Project applied case 1	20.33	205.89	206.98	1.09	0.11
Project applied case 2	20.70	205.89	207.49	1.60	0.16
Project applied case 3	20.98	205.89	207.90	2.01	0.20

Unit: million US$.

**Table 5:** Annual CO_2_ emissions for the residential building project.

Parameters	Value
Energy consumption (TOE/m^2^ · year)	0.018
Carbon emission factor (ton C/TOE)	0.812
Burning rate (%)	99
Subject project G.F.A (m^2^)	70,825
CO_2_ emission (ton CO_2_/year)	3,736.820

**Table 6:** The estimated values of guarantee value and CO_2_ emission reduction for each case.

Classification	Guarantee value (million US$)	Energy saving (%)	CO_2_ emission reduction (ton CO_2_/year)
Project applied case 1	0.11	33.96%	1269.02
Project applied case 2	0.16	46.96%	1754.81
Project applied case 3	0.20	50.16%	1874.39

**Table 7:** Total annual CER and payback period for guarantee value for each scenario of CER unit price.

	Case 1		Case 2		Case 3	
	Total CER (million US$)	Payback period (year)	Total CER (million US$)	Payback period (year)	Total CER (million US$)	Payback period (year)
Best scenario	0.065	1.64	0.090	1.78	0.096	2.11
Moderate scenario	0.037	2.86	0.052	3.09	0.055	3.67
Worst scenario	0.018	5.89	0.025	6.36	0.027	7.55
